# A Flexible Sensor Technology for the Distributed Measurement of Interaction Pressure

**DOI:** 10.3390/s130101021

**Published:** 2013-01-15

**Authors:** Marco Donati, Nicola Vitiello, Stefano Marco Maria De Rossi, Tommaso Lenzi, Simona Crea, Alessandro Persichetti, Francesco Giovacchini, Bram Koopman, Janez Podobnik, Marko Munih, Maria Chiara Carrozza

**Affiliations:** 1 The BioRobotics Institute, Scuola Superiore Sant'Anna, viale Rinaldo Piaggio 34, 56025 Pontedera (PI), Italy; E-Mails: n.vitiello@sssup.it (N.V.); s.derossi@sssup.it (S.M.M.D.R.); t.lenzi@sssup.it (T.L.); s.crea@sssup.it (S.C.); a.persichetti@sssup.it (A.P.); f.giovacchini@sssup.it (F.G.); carrozza@sssup.it (M.C.C.); 2 Biomechanical Engineering Laboratory, Institute for Biomedical Technology and Technical Medicine (MIRA), University of Twente, 7500 EA Enschede, The Netherlands; E-Mail: b.koopman@ctw.utwente.nl; 3 Laboratory of Robotics, University of Ljubljana, SI-1000 Ljubljana, Slovenia; E-Mails: janez.podobnik@robo.fe.uni-lj.si (J.P.); marko.munih@robo.fe.uni-lj.si (M.M.)

**Keywords:** distributed force sensor, wearable robotics, physical human-robot interaction, pressure-sensitive insole

## Abstract

We present a sensor technology for the measure of the physical human-robot interaction pressure developed in the last years at Scuola Superiore Sant'Anna. The system is composed of flexible matrices of opto-electronic sensors covered by a soft silicone cover. This sensory system is completely modular and scalable, allowing one to cover areas of any sizes and shapes, and to measure different pressure ranges. In this work we present the main application areas for this technology. A first generation of the system was used to monitor human-robot interaction in upper- (NEUROExos; Scuola Superiore Sant'Anna) and lower-limb (LOPES; University of Twente) exoskeletons for rehabilitation. A second generation, with increased resolution and wireless connection, was used to develop a pressure-sensitive foot insole and an improved human-robot interaction measurement systems. The experimental characterization of the latter system along with its validation on three healthy subjects is presented here for the first time. A perspective on future uses and development of the technology is finally drafted.

## 1. Introduction

In the last decade, human-centered robotics has seen huge developments in academic and industrial research, bringing to the market more and more robots able to cooperate safely with humans. Technological advancements and an increased interest by the medical community led to the development of service and domestic robots [1], medical robots for surgical aid [2] and rehabilitation [3–5], and wearable robots [6]. Wearable robots have been seen as a promising solution in many fields, including rehabilitation [4], functional replacement for disabled [7–11], walking assistance for the elderly [12] and load-carrying augmentation for soldiers [13].

A wearable robot, or exoskeleton, is an active robotic device that can be worn by the user. Links and joints of the exoskeleton are placed in correspondence of those of the human body, and are connected to the limbs in multiple points. These robots interact closely with the body and are supposed to cooperate actively with the user [12–17]. A distinctive characteristic of exoskeletons compared to other haptic robots is their closer physical and cognitive coupling with the user [17]. The mechanics and control that allow this physical and cognitive cooperation constitute the human-robot interface. In this work, we are interested to the physical human-robot interfaces, *i.e.*, the mechanical and sensor components that mediate the transfer of power between the user and the exoskeleton [18].

There are two main ways to connect wearable robots with the user: connection cuffs and orthoses. Connection cuffs are rigid links supporting a soft belt of adjustable size that is fastened around the user's limb. This solution has been used in both lower- [4,19] and upper-limb [20,21] exoskeletons. Orthoses, on the other hand, are shells made of plastic or other orthopaedic materials, potentially tailored on the user's limb, which are connected with the link of the robot. Orthotic shells, which have been also used in upper- and lower-limb devices [21–29], have the advantage of distributing the interaction evenly on a wide skin area, increasing the comfort and reducing the pain.

In both cases, getting an accurate measurement of the physical interaction is critical, as explained in [18] and [30–32], not only for control purposes, but also to assess the *reaction* of the user to the assistance given by the robot. Interaction force can be measured in two ways [33,34]: (i) by estimating the interaction torque through the measurement of the robot joint torque, as in the LOPES [19]; or (ii) directly measuring the force between exoskeleton and limb using load cells or similar technologies [32]. Both approaches have limitations. In the first method, the physical human-robot interaction (pHRI) force estimate can be corrupted by an inaccurate model of human-robot dynamics. In alternative to this, direct force measurements, while very accurate, have several drawbacks, *i.e.*, a multiple-degree-of-freedom exoskeleton would require as many localized force sensors as the number of contact points, leading to increased costs and complexity [33]; in addition, single-point measures such as those from a load cell hide all information related to the distribution of pressures at the cuff/orthosis: this can be extremely useful, being directly related with the safety and comfort felt by the user during the robot operation (high pressures might be uncomfortable or even painful to the user [35,36]); finally, single-point measures do not provide information about the preloading force when straps are used to fasten the limb to the device [33,37].

Based on the above considerations, over the last four years we developed a sensor technology for the measurement of distributed pressures, that we call Pressure Sensor Pads (PSP). We applied our technology on connection cuffs [34,37] of the lower-limb exoskeleton LOPES, and orthotic shells of NEUROExos [33], a powered elbow exoskeleton for physical rehabilitation [24,38]. Results from experimental activities showed that PSPs have good performances in terms of accurateness, sensitivity, dynamics behaviour and compliance with the mechanical requirements of the test platforms. This technology is based on an opto-electronic transduction principle, is completely modular and scalable, and allows to monitor pressures over areas of any sizes and shapes. This feature, along with the possibility to vary the pressure range given the specific application, makes our technology ideal to measure interaction forces in wearable robots. A further development of this technology was used to build a pressure-sensitive insole [39].

In this paper we survey the technology and its applications. Section 2 overviews the prototypes of the first (PSP1) and second (PSP2) generation of Pressure Sensor Pads, and introduces a novel PSP2 prototype for cuffs and orthoses-based on the same technology we developed for foot pressure monitoring. In Section 3, we give extended experimental results of our sensors applied to upper- and lower-limb exoskeletons. Section 3 also presents unpublished results obtained from the experimental validation of the new PSP2 prototype with the LOPES exoskeleton. Section 4 draws some conclusions and drafts a perspective on future uses and development of the technology.

## 2. Pressure-Sensor Technology

In this section we present the evolution of the design of the sensor, along with their mechanical and electrical characterization.

### 2.1. Design of the First Generation: PSP1.0 and PSP1.1

**Figure 1.**
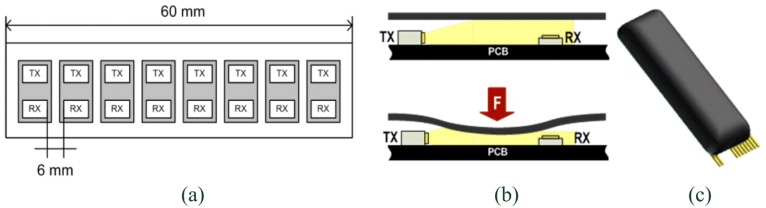
Overview of the PSP1: (**a**) specific scheme of the 1 × 8 array of sensitive elements of the PSP 1.0 and PSP1.1; (**b**) scheme of the transduction principle; (**c**) 3D design of the PSP1.1 (adapted from [37]).

The first generation of the pressure-sensor technology is loosely based on the Skilsens technology, a tactile technology conceived in our laboratory eight years ago [40,41]. The sensor is an optoelectronic pressure sensor made of two main parts: an external silicone bulk structure, and a printed circuit board (PCB) which houses an array of sensitive elements. The dimension of the sensor can be adjusted based on the constraints given by the specific application. In this example, the size is 20 × 60 mm which allows to house an array of 1 × 8 sensitive elements. The technology can be applied to custom sizes and number of sensors. Each sensitive element is composed of a light transmitter, a LED (an InGaN chip technology, high luminosity green LED, OSA Opto Light GmbH, Berlin, Germany) and a receiver, a photodiode (an analog ambient light opto-electronics transducer with current output, Avago Technologies Ltd., Singapore). The silicone bulk covers the electronics components and plays an active role in the transduction principle: when a load is applied on the sensor, the cover deforms itself, the light is screened and the sensor changes proportionally its output voltage (see Figure 1).

By varying the silicone material and the thickness of the cover, the structural rigidity of the sensor can be changed. Two different covers were developed for two different applications (see Section 3).

The first cover (named PSP1.0) has a bulk made of two different rubbers, an extern black silicone (ECOFLEX, Shore 00-30, Smooth-On Inc., Easton, PA, USA) and an internal transparent and less stiff silicone (ECOFLEX, Shore 00-15, Smooth-On Inc.). The total thickness of the pad is 8 mm [33]. Only the external silicone obstructs the light path and induces a change of the sensor's output. The second cover (named PSP1.1) has only a single silicone shell (Sorta Clear 40, Shore 40 A, Smooth-On Inc.) coloured with black pigment, for a total thickness of the sensor of 7 mm [37] (see Figure 1). The silicone Sorta Clear 40 was characterized by Axel Products Inc. (Ann Arbor, MI, USA) to define their basic elastomeric properties through the execution of four mechanical tests [42,43]: simple tension (using a long and thin specimen and a laser extensometer), pure shear (using a wide specimen and a laser extensometer), equal biaxial stress (using a circular specimen stretched on radial direction, and a laser extensometer), and volumetric compression (using a cylindrical specimen). All of the tests were performed under slow cyclical loads to avoid the Mullin effect (changing structural properties during the first loading cycle). Data of characterization were used to create a nine-parameter Mooney-Rivlin solid model, necessary to develop a 3-dimensional finite-element (3D FE) model of the silicone cover in ANSYS 12 (Ansys Inc., Canonsburg, PA, USA).

**Figure 2.**
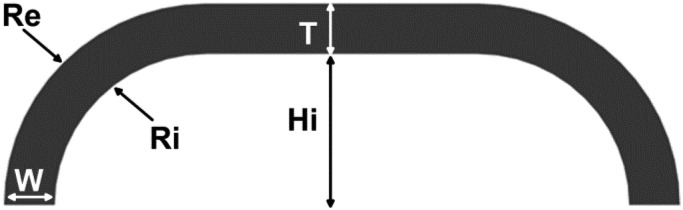
Cross section of the PSP1.1 (adapted from [37]).

It is possible to vary the sensing range of the sensor by changing some design parameters. In the case of PSP1.0, force range can be adjusted by changing the thickness of the internal silicone layer. In the case of PSP1.1 the desired force range can be changed by adjusting the five geometrical parameters that characterize the section of the silicone cover: (i) the internal height Hi, (ii) upper-part thickness T, (iii) the basis thickness W, (iv) and (v) the internal and external radii which connect the basis to the upper part, Ri and Re (see Figure 2).

While the design of PSP1.0 resulted from an heuristic experimental process, the silicon cover of PSP1.1 was designed thanks to a 3D FE analysis by using ANSYS 12. Given a desired sensitive range of 60 N at a deformation of about 1.5 mm, which leads to saturation of the sensor's output, many simulations were carried out with different sets of geometrical parameters Hi, T, W, Ri and Re.

**Figure 3.**
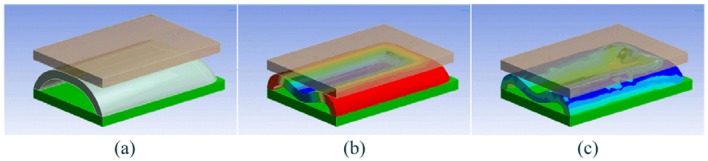
Finite element simulation of PSP1.1: (**a**) un-deformed structure; the rigid flat indenter is transparent brown, the silicone structure is grey and the PCB is green; (**b**) total deformation representation; (**c**) total stress representation (adapted from [37]).

In each simulation a rigid flat indenter pushed on the sensor (see Figure 3) with an increasing load onto the top face of the sensor (Figure 3). The contact region was modelled as a rigid friction connection; this choice was based on the difficulty to model the friction on hyper-elastic material [44]. On the other hand, the contact area between the silicone bulk and the PCB was modelled as fixed support. We simulated the load by imposing a displacement of the indenter with respect to the PCB, and for each deformation state we evaluated the total stress state, the deformation state and the total force response of the structure. The FE analysis shows that the structure suffers of a sinking effect, which increases the light occlusion for small loads. Although it would increase the sensitivity of the sensor for low forces, at the same time, it would also lead to a reduction of the sensing range: smaller load would cause the silicone cover to touch the PCB and, as a consequence, the sensor to saturate.

The geometrical parameters leading to a desired interaction force of 60 N, corresponding to an average pressure on the pad of 50 kPa (a desired force range chosen based on a series of preliminary experiments [34]), are: Hi = 4 mm, T = 3 mm, W = 3 mm, Ri = 6 mm and Re = 6 mm. By using these parameters, the silicone cover was obtained by casting liquid silicone in a male/female acrylic mold. After the polymerization the silicone cover was glued on the PCB.

### 2.2. Experimental Characterization of PSP1.0 and PSP1.1

For both PSP1.0 and PSP1.1 an experimental characterization was carried out to find their structural (force/deformation) and opto-electrical (sensor's output/force) behaviour [33,37]. Both characterizations were obtained by means of a similar procedure, *i.e.*, by applying a load on the sensor using a rigid flat indenter, like in the FE simulations, while recording sensor deformation and output voltage. The characterizations were performed using an INSTRON 4464 testing machine (INSTRON Inc., Norwood, MA, USA), equipped with a 1 kN load cell. For each sensor five loading-unloading cycles were performed at a relatively low speed (0.1 mm/min for PSP1.0, and 1 mm/min for PSP1.1), to simulate a quasi-static load.

**Figure 4.**
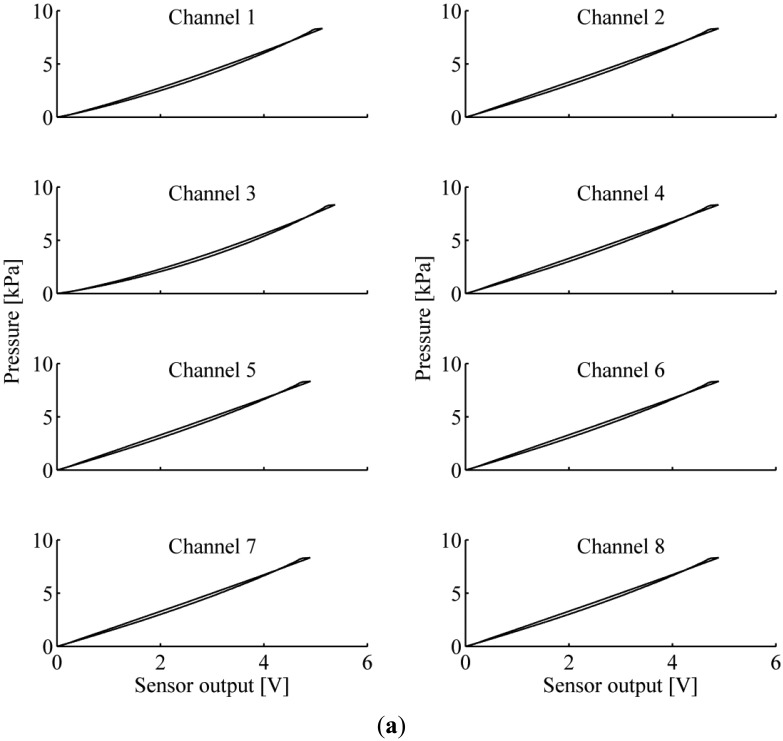

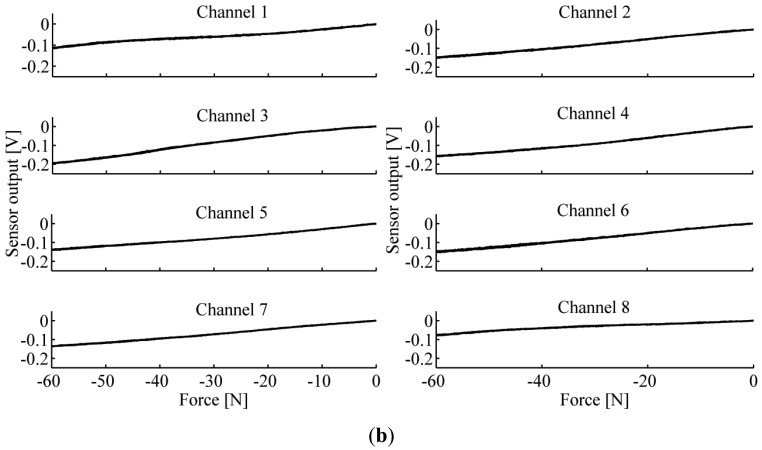
Force (or pressure) *vs.* output voltage of the PSP1 1 × 8 array of sensitive elements: (**a**) PSP1.0; (**b**) PSP1.1 (adapted from [33,37]. (a) is a slightly adapted reprinted graphics from [33], ©2011, with permission from Elsevier).

In order to know the specific response of the sensor to the applied load, we characterized the voltage output of the sensor against the applied load. For each sensor output, experimental data were fitted with a second-order polynomial function to obtain a model of the sensor. Figure 4 shows the output voltage *vs.* applied force behaviour of each couple of light emitter-receiver of the 1 × 8 array of sensitive elements of both PSP1.0 and PSP1.1. A summary of the main technical characteristics of the sensors are reported in the Table 1.

Table 1 shows that the maximum loading force on the surface (equal to 12 cm^2^) of PSP1.1 is greater than the one of PSP1.0, whereas the maximum deformation is comparable, and the maximum hysteresis slightly decreases. The latter difference is mainly due to the absence in PSP1.1 of the viscous internal layer of transparent silicone. Despite a different force range, PSP1.0 and PSP1.1 have a comparable force vs. output voltage behaviour: voltage-to-force curves increase monotonically and have small hysteresis compared to the full-scale range.

### 2.3. Design of the Second Generation: PSP2.0 and PSP2.1

The transduction principle of the second generation (PSP2) is conceptually analogous to the principle described for the first generation. The main differences are: (i) the structure of the sensor, which is composed by independent silicone cells, one for each couple of light emitter-receiver, and (ii) the shape of the silicone cover.

**Figure 5.**
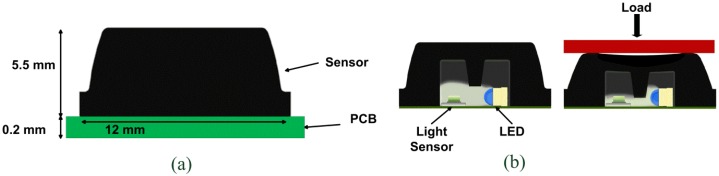
Overview of the second-generation PSP sensitive element: (**a**) dimension of the sensitive element, (**b**) transduction principle (adapted from [39], ©2011 IEEE. Reprinted with permission).

Thus, each sensitive element is composed of two main parts: the silicone cover and a PCB (which can be either flexible or rigid) that houses the sensitive component. The sensitive element is composed of the light emitter, a high luminosity green LED (an InGaN chip technology, high luminosity green LED, OSA Opto Light GmbH), and the light receiver, a photodiode (an analog ambient light opto-electronics transducer with current output, Avago Technologies Ltd.). The LED faces the near receiver. The cover is realized with a silicone shell coloured by a black ink, and has the shape of a pyramidal frustum with a square basis, with an internal central curtain (see Figure 5). The dimension of the frustum base is 12 × 12 mm^2^, while the top face is 10 × 10 mm^2^, and the height is 5.5 mm. Figure 5(b) points out the transduction principle: when a load is applied onto the frustum top face, the silicone bulk deforms itself, the curtain within the cover closes the light way, and the sensor varies its output voltage [45,46]. This contact surface provides a 1 cm^2^ resolution to the estimation of pressure distribution.

**Figure 6.**
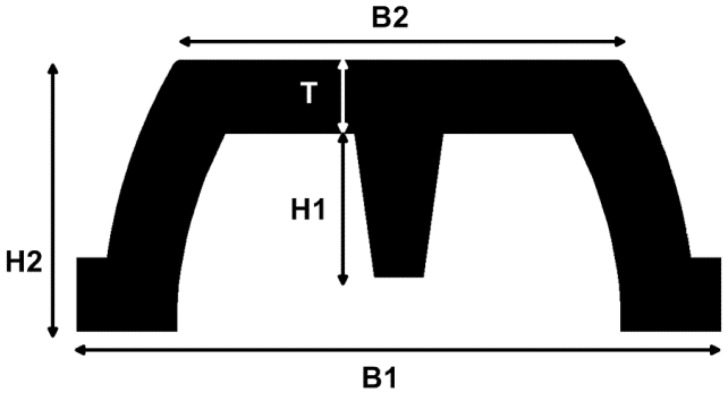
Cross section of the PSP2 silicone cover; the geometrical parameters are: cover thickness T, height of the curtain H1, pyramidal frustum height H2, square base size B1, square top-face size B2.

Figure 6 shows a cross section of the PSP2 silicone cover. The shape of the cover is identified by five geometrical parameters: (i) thickness T, (ii) the height of the curtain H1, (iii) the height of the pyramidal frustum H2, (iv) the side of the base B1, (v) the side of the top-face base B2. The value of these parameters and the material have to be chosen in order to obtain a shell sensible to the load applied.

For the second generation we realized two different silicon covers, for two different prototypes: PSP2.0 and PSP2.1, made with different silicones and having two different sets of geometrical parameters. For PSP2.0 we used Dragon Skin 10 Medium (Smooth-On Inc., Shore 10 A), whereas for PSP2.1 we used the stiffer Sorta Clear 40 (Smooth-On Inc., Shore 40 A). Similarly to the silicone used for the cover of PSP1.1, the Dragon Skin 10 was characterized by the Axel Products Inc., to define its basic elastomeric properties [42,43], and data were used to define the nine-parameter Mooney-Rivlin solid model to create the 3D FE model in ANSYS 12.

**Figure 7.**
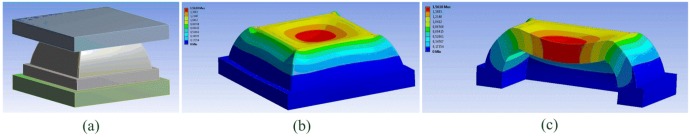
3D FE simulations of PSP2.1: (**a**) simulation environment: in blue the rigid indenter, in grey the silicone structure, in green the PCB; (**b**) map of the total deformation, (**c**) cross-section of the pyramidal frustum showing the sinking effect.

Similarly to PSP1.1, PSP2 geometrical parameters were identified by iterative simulations in which a rigid flat indenter parallel to the PCB applied an increasing load onto the top-face of the pyramidal frustum (see Figure 7). The final choice of the geometrical parameters was determined by the need of minimizing the sinking effect of the top face (see Figure 7(c)). Geometrical parameters were finally selected to address the requirements of two different applications.

For PSP2.0, geometrical parameters were selected with the objective to develop an insole made of an array of sensors to measure the foot-ground interaction pressure during gait. The pressure range of the sensitive element was set to 0–500 kPa. This pressure range ensures to measure, without saturations, the vertical ground reaction force (vGRF) during gait of a standard man (about 70 kg), walking at a normal speed (up to 1.3 m/s). A typical vertical force pattern shows, in fact, that the value of the peak occurring in response to the weight accepting event, is approximately the 130% of the body weight [47], that is here considered to be applied in one third of the sensitive area. The geometrical parameters of PSP2.0 are: T = 3 mm, H1 = 2.3 mm, H2 = 5.5 mm, B1 = 12 mm, B2 = 10 mm.

On the other hand, PSP2.1 silicone pyramidal frustum was designed with the final aim of developing an array to monitor the interaction force at a cuff of the lower-limb exoskeleton LOPES, and therefore the sensing range was set to 3.5 N. This value corresponds to a maximum pressure of about 35 kPa, which is comparable with the pressure range explored in [37]. The geometrical parameters of PSP2.1 are: T = 1.5 mm, H1 = 2.9 mm, H2 = 5.5 mm, B1 = 12 mm, B2 = 9 mm.

Similarly to PSP1, PSP2 silicone covers were obtained by casting liquid silicone in acrylic molds. After polymerization, silicone covers were glued onto the PCB.

### 2.4. Characterization of the Second Generation

As for the first generation, the experimental characterization aimed at assessing the force *vs.* deformation behaviour of the silicone cover, as well as at constructing the force- (or pressure)-to-output voltage curve of each sensor.

The force-to-output voltage characterization of PSP2.0 and PSP2.1 was performed by using a 3-axial platform (TAP) machine, developed at The BioRobotics Institute of Scuola Superiore Sant'Anna (Pisa, Italy), equipped with a six-axis load-cell (ATI Nano-17 SI-25-0.25, ATI Industrial Automation, Apex, NC, USA), and a rigid flat indenter. While applying the deformation on the sensitive element (setting a maximum deformation of 1.2 mm), we recorded the reaction force and the output voltage of each sensor.

**Figure 8.**
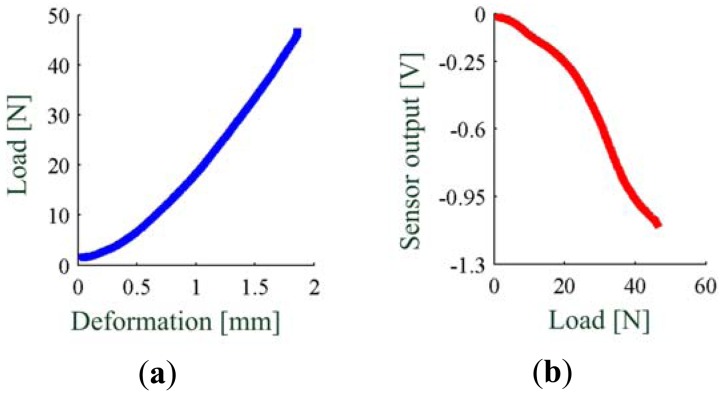
Characterization of the sensitive element of PSP2.0: (**a**) quasi-static force-to-deformation characterization; (**b**) quasi-static force-to-voltage curve (adapted from [39], ©2011 IEEE. Reprinted, with permission).

For PSP2.0 we performed a quasi-static force vs. deformation characterization, executing three loading-unloading cycles with a loading speed of 0.084 mm/s (*i.e.*, ∼5 mm/min). All data were off-line low-pass filtered with a third-order Butterworth filter, with cut-off frequency equal to 100 Hz (Matlab^®^*filtfilt* function). Force-to-output voltage loading-unloading cycles were fitted by a third-order polynomial function which was found to be the best compromise in terms of complexity and goodness of fit, with root mean square error (RMSE) equal to 0.045 N. The maximum load generates a vertical deformation of about 1.8 mm (see Figure 8(a)). The non-amplified output voltage of the sensor has a dynamic range of about 1.1 V, corresponding to a 50 N load on the sensor (see Figure 8(b)) [39].

**Figure 9.**
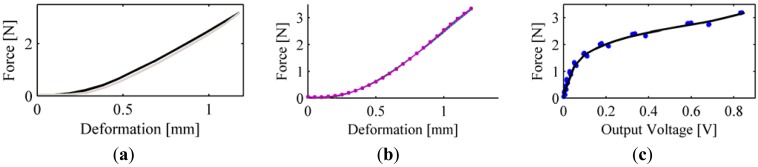
Results of the PSP2.1 characterization: (**a**) quasi-static force-to-deformation loading-unloading, averaged over three iterations (black line is the loading phase, grey line is the unloading phase); (**b**) all fitting curves of loading-unloading cycles at seven different levels of loading speed (namely, 0.05, 0.1, 0.2, 0.3, 0.4, 0.5, 1 mm/s); (**c**) quasi-static force-to-voltage curve (blue dots are experimental data, black line is the smoothing spline).

For PSP2.1 we executed three loading-unloading cycles with a loading speed of 0.1 mm/s to get a quasi-static force vs. deformation characterization. Then, in order to assess the mechanical hysteresis we also performed loading-unloading cycles at seven increasing levels of loading speed, *i.e.*, from 0.05 mm/s to 1 mm/s (three cycles for each speed). All data were off-line low-pass filtered with a third-order Butterworth filter, with a cut-off frequency equal to 30 Hz (Matlab^®^*filtfilt* function). Data reported in Figure 9(a) shows that the silicone cover has a non-linear force-to-deformation behaviour in quasi-static condition, with a slight hysteresis of 0.16 N, *i.e.*, 5% of the full-scale range. All force-to-deformation loading-unloading cycles were fitted by a fourth-order polynomial function. In Table 2 we report the RMSE and the R^2^ of the fitting, as well as the loading-unloading hysteresis. Finally, Figure 9(b) reports the fitting curves for all loading speeds.

The force-to-output voltage characterization of the sensor is reported in Figure 9(c). The output voltage has a monotonic rising trend, and ranges from 0 to 0.85 V. Quasi static force-to-voltage curve is well fitted by a smoothing spline (Matlab^®^*cftool*), with RMSE = 0.0958 N.

## 3. Applications

This section provides an overview of the scenarios in which the PSPs (of both first and second generations) were tested. Firstly, we will report the results from the test of PSP1.0 with the exoskeleton of upper-limb NEUROExos and from the test of the PSP1.1 with the cuffs of the lower-limb LOPES exoskeleton. Secondly, we will show the results of the application of the PSP2.0 and PSP2.1 respectively for the construction of a pressure-sensitive insole and a novel prototype of sensorized cuff of the LOPES exoskeleton.

All experimental tests were carried out with healthy subjects.

### 3.1. Application of PSP1.0 to the Elbow Powered Exoskeleton NEUROExos

In this application we wanted to assess the usability of the PSP1.0 to measure the interaction pressure between the elbow exoskeleton NEUROExos and the user forearm surface, as well as to discriminate any user *reaction* to the robot action during a prototypical rehabilitation task. In particular, we were interested to discriminate between two conditions:
Case 1)“*no action*”: the user is passive and does not perform any voluntary elbow muscle activation; the robot moves user's elbow along a sinusoidal reference trajectory;Case 2)“*pre-defined action*”: the user is asked to simulate a reaction to the robot action by voluntary changing (*i.e.*, increasing or decreasing) one of the reference motion features (*i.e.*, sinewave frequency or amplitude).

**Figure 10.**
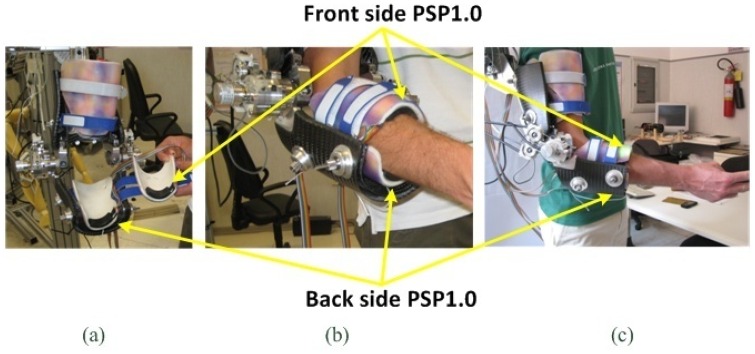
The NEUROExos platform equipped with two PSPs 1.0. (**a**) PSP placement onto inner-side of exoskeleton inner-shells; front (**b**) and lateral (**c**) view of a subject wearing the NEUROExos (adapted from [33], ©2011, with permission from Elsevier).

The NEUROExos is an active orthosis for the rehabilitation of the elbow, able to transmit torques to the user's elbow. Its flexion-extension rotation axis is endowed with a 4-DOF passive mechanism which ensures automatic alignment between the human and the robot rotation axis [38]. NEUROExos has double-shelled links and an actively adjustable passive-compliance actuator, that together with the 4-DOF mechanism allow a highly ergonomic and safe physical interaction between the subject's arm and the orthosis (Figure 10(a)) [24]. The outer shell of each link is made of carbon fibre and transmits the force to the arm segments through inner shells made of a flexible polymeric material. The inner shells are composed of two valves and tailored on the subject arm surface to maximize the human-robot contact area and to improve the comfort reducing the pressure on the skin (Figure 10).

For the proposed experiment, two PSPs 1.0 were placed between the forearm and the inner border of the polymeric inner shells (see Figure 10(a)). PSPs were placed as close as possible to the hand, to minimize the motion artefacts due to the skin/muscle shape changes during motion. Each PSP had its own acquisition channel and cabling. They were acquired and processed by a real-time processing unit (NI PXI-8196 RT, National Instruments Corporation, Austin, TX, USA) equipped with a multifunction data acquisition card (M-series, NI PXI-6259, National Instruments Corporation). The sensor signals (8 analog signals for each PSP) were sampled at 50 kHz, low-pass filtered with a moving average over 50 samples (with zero overlapping) and de-sampled at 1 kHz. The pressure measured by the eight sensitive elements of each PSP was averaged to obtain the mean pressure acting on the PSP [33].

**Figure 11.**
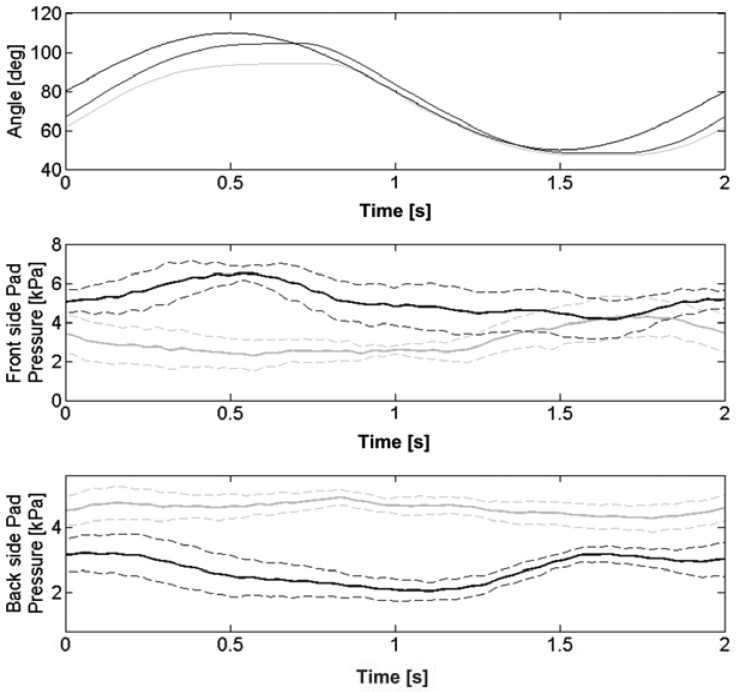
NEUROExos joint trajectory, front- and back-side PSP1.0 pressure profiles during a prototypical rehabilitation task, with and without the user *reaction*. Top panel reports: the reference trajectory of the rehabilitation task (dashed line), the “no action” trajectory (gray line), and the “pre-defined action” performed by the subject (black line). Middle and bottom panels report respectively front- and back-side PSP1.0 pressure profiles: the “no action” condition is the gray line, the “pre-defined action” is the black line. Pressure profiles are averaged over ten sinusoidal motions and reported along with standard-deviation contour (dotted line). We assume that the elbow is fully extended when the joint angle is equal to zero (figure adapted from [33], ©2011, with permission from Elsevier).

The result of this experiment was that PSP1.0 was able to record pHRI pressure and to detect the user *reaction*. For instance, this is evident from Figure 11. In this case NEUROExos was programmed to displace the user elbow joint along a 30-deg sinusoidal flexion-extension trajectory with frequency equal to 0.5 Hz. Figure 11 compares the joint trajectory and the PSP average pressure profiles (of front- and back-sides PSPs) of the two conditions “no action” and “pre-defined action” (in this specific example the user was asked to increase the sine wave pace). Indeed, reported data points out that user action results in different PSP pressure profiles: the user tries to anticipate the movement of the robot and generates a higher interaction force/torque in the same direction of the movement, *i.e.*, higher pressure on the front- and back-side PSPs during respectively the elbow flexion and extension phases.

### 3.2. Application of PSP1.1 to the Lower-Limb Exoskeleton LOPES

In this application we wanted to assess the usability of the PSP1.1 to measure the pHRI pressure between the user and a lower-limb exoskeleton, and to compare the pressure estimated by the PSP1.1 with the output of a classical load cell.

**Figure 12.**
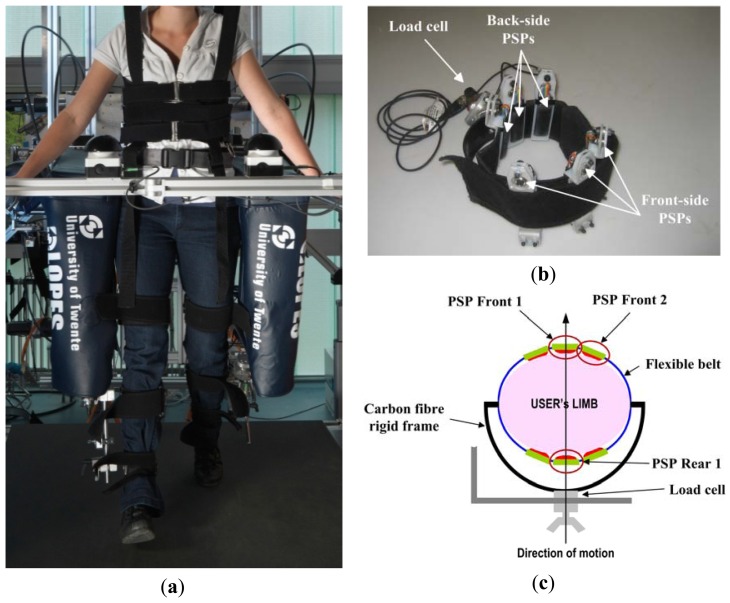
(**a**) Overview of the LOPES exoskeleton; (**b**) right-leg thigh cuff sensorized with six PSPs 1.1; (**c**) schematic view of the right-leg sensorized cuff (adapted from [37]).

The LOPES is a lower-limb exoskeleton for gait training. It has three DOFs for each leg, two at the hip (*i.e.*, flexion-extension and abduction-adduction) and one at the knee, and two DOFs to move the pelvis in the coronal and horizontal planes. LOPES joints are actuated by series elastic actuators capable of applying low-impedance torque profiles onto the corresponding human articulations and to implement virtual impedance fields [19,48]. The LOPES interfaces the human subject limbs through commercial cuffs (Hocoma AG, Volketswil, Switzerland). In particular, one cuff interfaces the thigh, and two cuffs interface the lower leg. Each cuff is realized by a flexible belt connected to a c-shape frame made of carbon fibre, which is ultimately linked to the robot linkages through steel bars (see Figure 12(a–c)).

In this experiment we equipped the right-leg thigh cuff with six PSPs placed between the leg and the belt. The six PSPs were fixed to the belt with their longitudinal axes parallel to the longitudinal axis of the limb: three PSPs were placed onto the front-side surface of the cuff, and three ones on the back side (see Figure 12(b,c)).

PSPs signals were acquired using a 32-channel DAQ board, with a sampling frequency of 2 kHz and digitally filtered with a fourth-order Butterworth filter with a cut-off frequency of 40 Hz. Furthermore, the attachment point of the cuff to the LOPES linkage was sensorized with a 6-axis load cell (ATI Mini 45, ATI Industrial Automation) to provide a measurement reference for assessing the reliability of the PSP outcome (see Figure 12(b)).

One subject walked on a treadmill within the LOPES at a constant speed of 4 km/h for about 250 gait cycles in two different conditions:
Case (1)*transparent*: the LOPES was controlled in zero-torque mode [49], *i.e.*, it operated as transparent as possible;Case (2)*viscous field*: we applied a virtual viscous field of 10 Nm/rad·s^−1^ at the LOPES hip flexion-extension joints; the viscous field simulated a typical resistive gait training task.

**Figure 13.**
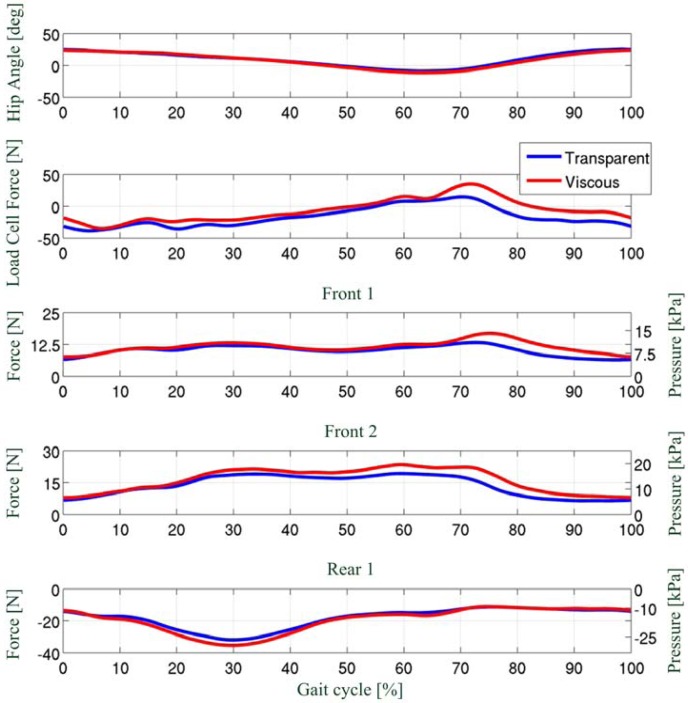
Test of PSP1.1 on the LOPES: profiles of right hip flexion-extension angle, total interaction force measured by the load cell, and the force estimated by three of the six PSPs (*i.e.*, “Front 1”, “Front 2”, “Rear 1”). Data are shown for two conditions: transparent and viscous field (figure adapted from [37]).

Signals from each PSP were combined to estimate the total force acting on the pad. Force profiles from each PSP were than averaged over gait cycles. Figure 13 reports, for one of the subjects, the right hip angle, the total interaction force measured by the load cell, and the force estimated by three of the six PSPs (two PSPs placed onto the frontal side, namely *Front 1* and *Front 2*, and one PSP placed onto the back side, namely *Rear 1*, see Figure 12(c)) for the two above conditions (the common x-axis represents the percentage of the gait cycle). The beginning of the cycle (0 or 100%) corresponds to the foot impact on the ground. The stance phase ranges from 0 to about 50–60% of the cycle, where the toe-off takes place. The remaining part of the cycle (60–100%) corresponds to the leg swing phase. For sake of clarity, it is worth mentioning that the output of the load cell is positive when the net interaction force is higher on the front-side surface of the cuff and, *vice versa*, it is negative when the net interaction force is higher on the back-side of the cuff surface.

Data reported in Figure 13 show two main interesting results. First, all PSPs record higher peak force in the *viscous field* condition. This is coherent with the fact that in the *viscous-field* condition the LOPES is less transparent and has a higher loading effect on the user gait. Second, there is a clear discrepancy between the output of the load cell, which represents the overall interaction force, and that of the PSPs. Indeed, in the central part of the mid stance (10–50% of the gait), while the resultant net force measured by the load cell is almost null, all PSPs record an increase of the interaction force. These peaks, *that would not have been detected by the load cell*, are much likely due to the co-contraction of the leg muscles during the stance phase, and to the consequent change in the shape and size of the thigh.

In conclusion, this experiment showed that PSP1.1 were a suitable solution to monitor the pHRI force in a lower-limb exoskeleton, and, furthermore, provided additional information compared to localised force measurements through classical six-axis force sensors.

### 3.3. Application of PSP2.0 to Gait Analysis

PSP2.0 sensitive element was used to conceive and develop a pressure-sensitive foot insole to allow biomechanical assessment of gait [39].

**Figure 14.**
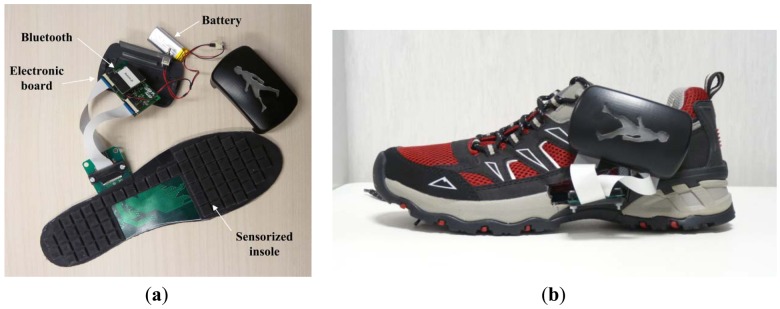
PSP2.0-based pressure-sensitive insoles. (**a**) Overview of the pressure-sensitive insole on the bench; (**b**) two pressure-sensitive insoles integrated into normal sneaker shoes.

Each insole is made of an array of 64 sensitive sensors for the measurement of the pressure over the plantar area (with the exception of the plantar arch), and an electronic board that processes and transmits wirelessly the data, sampled at 100 Hz, to a remote data logging computer via a Bluetooth connection. The developed sensorized insoles can fit into a normal sneaker shoes of EU size 42 and run continuously for up to 7–8 hours with an on-board battery. Figure 14 reports an overview of the system.

Pressure-sensitive insole voltage signals are online converted into pressure values through a pre-computed calibration function and a Laplacian surface of smoothing is applied to the pressure map to remove pressure outliers and regularize the surface. The pressure map is used to extract the values of the vertical ground reaction force (vGRF), the position of the centre of pressure and the partial forces on the foot tip and heel. These variables, together with their first-order time derivatives, are of high interest in gait analysis and have been used to develop an automated gait segmentation algorithm using a common machine learning technique, Hidden Markov Model [50,51].

**Figure 15.**
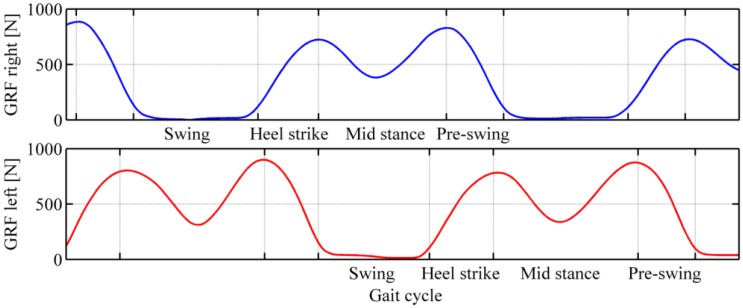
Gait phases recognition through the pressure-sensitive insole for both left (top panel) and right (bottom panel) feet.

As an example of the performance of the pressure-sensitive insoles, Figure 15 reports the variation of the vGRF of both feet during the gait. For both feet, it is possible to distinguish the principal phases of the gait [47], that are: (i) the contact of the foot with the floor, corresponding to the increasing of the vGRF until the first peak, (ii) the mid-stance phase, corresponding to the variation of vGRF until the second peak, (iii) the pre-swing phase, corresponding to the decreasing of the vGRF, and (iv) the swing phase, zero force on the insole.

### 3.4. Application of PSP2.1 to the Lower-Limb Exoskeleton LOPES

The objective of this application was to assess the usability of the new PSP2.1-based LOPES sensorized cuff to measure the pHRI force between the user limb and the exoskeletal attachment points.

**Figure 16.**
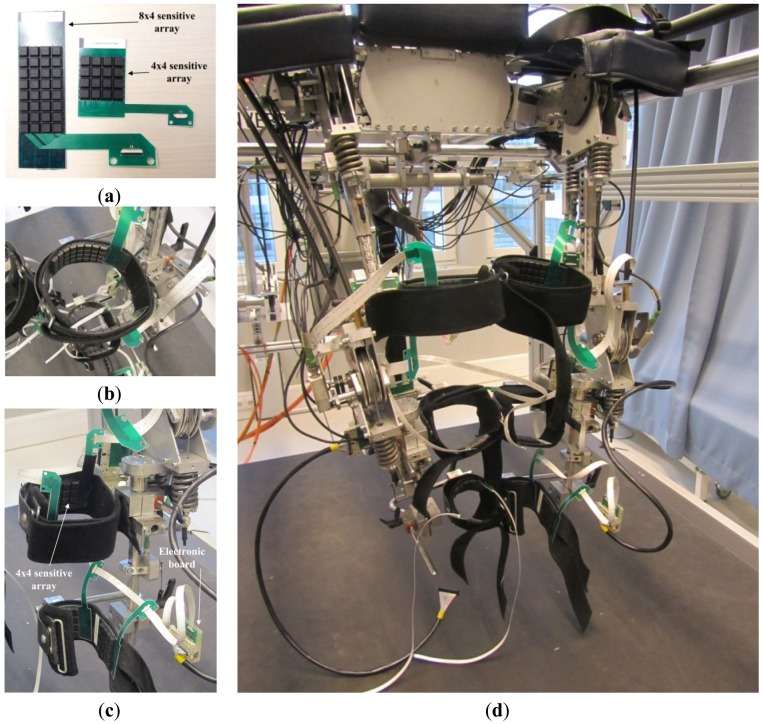
New PSP2.1-based LOPES sensorized cuffs. (**a**) Overview of the 8 × 4 and 4 × 4 sensitive arrays; (**b**) sensorized thigh cuff; (**c**) sensorized shank and ankle cuffs; (**d**) overview of the LOPES with all of the six cuffs endowed with PSPs 2.1.

In this application scenario, all of the six LOPES cuffs were sensorized with two PSP2.1 arrays (see Figure 16). Thigh cuffs were covered with two 8 × 4 sensitive arrays, one for the front- and one for the back-side surface of the cuff. Shank and ankle cuffs were covered with two 4 × 4 sensitive arrays. As for the pressure-sensitive insoles, data from cuffs were collected, processed and wirelessly transmitted to a remote PC by a custom electronic board. Through the custom electronic board, data from each cuff were sampled at 1.8 kHz, low-pass filtered and de-sampled at 100 Hz, and finally transmitted to the remote PC through a Bluetooth connection. Electronic boards were fixed on the exoskeleton using a custom box, which houses the electronics, the Bluetooth transmitter and the battery (Figure 16).

To test the usability of the new sensorized cuffs, we performed the following experimental protocol. Three subjects walked on a treadmill within the LOPES, in three different conditions:

1. the subject walked at a constant speed of 2.5 km/h with an assistive torque for the hip flexion-extension (all other LOPES joints were controlled in zero-torque mode);
2. the subject walked at a constant speed of 4 km/h with an assistive torque for the hip flexion-extension (all other LOPES joints were controlled in zero-torque mode);
3. the subject walked at a constant speed of 4 km/h without any assistive torque (all LOPES joints were controlled in zero-torque mode).

Assistive torque to hip flexion-extension was provided by means of the adaptive assistive algorithm based on the use of motor primitives proposed in [52].

**Figure 17.**
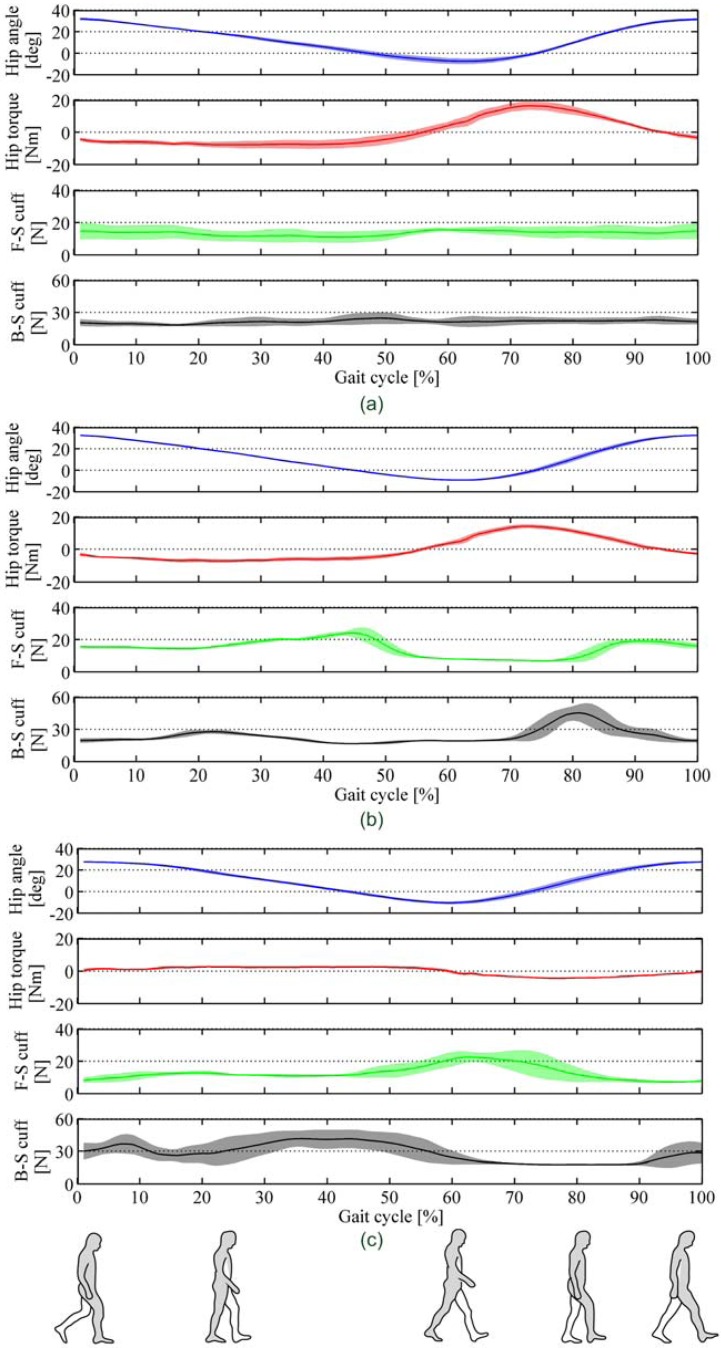
Hip kinematic and dynamic variables for Subject #1: right-leg hip flexion-extension joint angle and torque, and total force recorded by front- (F-S) and back-side (B-S) thigh-cuff PSPs. Data are averaged over 20 gait cycles (solid line), and shown along with the standard deviation contour (shadowed), for three conditions: (**a**) gait velocity is 2.5 km/h, with assistive torque; (**b**) gait velocity is 4 km/h, with assistive torque; (**c**) gait velocity is 4 km/h without assistive torque.

For each (front- and back-side array) we computed the total force applied on it. For each of the above three conditions, Figure 17 reports, for one of the subjects, the following data averaged over 20 gait cycles: right-leg hip flexion-extension angle and torque, total force recorded by the front- and back-side PSP arrays of the right-leg thigh cuff. In Figure 17 mean profiles (solid coloured lines) of all variables are reported with standard deviation (shadowed contours) along the gait cycle, expressed in percentage.

Table 3 summarises for all of the three subjects the mean and maximum force values (averaged over 20 gait cycles) recorded by the PSPs of the right-leg thigh cuffs.

Results reported in Figure 17 and Table 3 point out that the PSPs actually record three different pHRI force patterns, for the three different conditions. Indeed, in the case of gait velocity equal to 2.5 km/h, front- and back-side PSPs measure a slightly changing value of force along the gait cycle. Furthermore, for all subjects, the maximum and the average values of force applied on the PSPs are the lowest ones, between the three gait conditions. This result is in line with results achieved in previous works [52]: this level of assistance, at this gait speed, renders the LOPES more transparent, thus reducing the interaction force. Indeed, the force recorded by the PSPs represents the preloading force for fastening the cuff around the user limb. On the other hand, in the case of gait velocity equal to 4 km/h the PSP force profiles vary along the gait cycle: two different patterns can be recognized depending on the presence/absence of the torque assistance. When the assistance is on, the front-side pad shows a decreasing of the interaction force between the toe-off and the middle-swing phases (50–80% of the gait cycle), while the back-side pad measures a peak in the middle of the swing phase (around 80% of the gait cycle). This is mostly due to the action of the assistive torque in the flexion direction. It is also worth noting that the peak of the interaction force recorded by the back-side pad is delayed about 10% of the gait cycle compared to the peak of the assistive flexion torque (around 70% of the gait cycle). This delay is due to the pHRI dynamics: human limb and robot link are not rigidly connected: before the torque is actually transmitted from the robot to the user, muscle and other soft tissues must be squeezed by the cuff, thus generating a delay in the mechanical transmission. When the assistance is off, both the front- and back-side pads show a peak force in correspondence of the maximum joint angle acceleration, respectively at the end of the stance phase (60% of the gait cycle) and at the end of the swing phase (0% of the gait cycle). This pattern is well explained by the fact that—in this case—since no assistance is provided by the robot, the subject transfer mechanical power to the robot to accelerate/decelerate its linkages.

## 4. Conclusions

In this paper we overviewed the evolution of the pressure-sensitive technology developed at Scuola Superiore Sant'Anna, based on an opto-electronic transduction principle which allows to monitor pressure distribution at the pHRI surface, as well as at the foot-ground interface.

The presented works show the importance of the silicon cover for the proposed technology: it plays a crucial role in the transduction principle, and enhances the scalability of the PSPs in terms of: (i) overall size of the sensitive area (we passed from the 20 × 60 mm^2^ of the PSP1 to the 12 × 64 mm^2^ if the PSP2.0 used for the pressure-sensitive foot insole); (ii) number of sensitive elements, (iii) pressure sensitive range, that we explored from 8.3 kPa (in the case of PSP1) up to 500 kPa for the sensorized insole.

In all application scenarios PSPs showed relevant performance and validated the proposed approach to monitor the pHRI pressure/force, despite the non-ideal non-uniform load distribution on the top surface of the silicon cover (being the non-uniform load distribution a result of the interaction of the PSP with a curved surface, such as the one of limb segments or the sole). These results confirmed the capability of the technology to estimate with good accuracy the load also when interacting with a curved indenting surface, as early demonstrated by bench tests in [37], provided that its curvature radius is sufficiently high compared to the size of the top surface of the PSP silicone cover. Furthermore, successful use of the proposed technology was possible also thanks to: (i) the possibility to assemble the PSP on a flexible PCB (this is the case of the PSP2.0 and PSP2.1 arrays), (ii) the wireless connection, (iii) the low-power consumption (in the case of the insole the maximum requested power is about 0.5 W), and (iv) the inherent cost effectiveness of the technology.

Future works aim at exploiting the proposed technology and its application examples in intensive applications in both laboratory and clinical settings. For instance, the sensorized insole will be used to monitor the gait of lower-limb amputees to provide them with an augmenting proprioceptive feedback from the missing limb [53]. In addition, a sensorized mat with more than four thousands of PSP2.1 sensitive elements will be used to monitor the posture of preterm infants during the rehabilitation process, similarly to what has been proposed recently in [54,55].

## Figures and Tables

**Table 1.** Main features of the PSP1 prototypes: maximum loading force, maximum pressure, maximum deformation, average stiffness, and maximum hysteresis expressed in percentage of the full-scale range of the input (values are extracted from [33,37]).

	**PSP1.0**	**PSP1.1**
**Maximum loading force**	10 N	60 N
**Maximum pressure on the surface**	8.3 kPa	50 kPa
**Maximum deformation**	1.9 mm	1.5 mm
**Average stiffness**	5.26 N/mm	40 N/mm
**Hysteresis**	3.8%	3%

**Table 2.** RMSE, R^2^ and maximum hysteresis for force-to-deformation characterization of PSP2.1.

	**Quasi-static**	**0.05 mm/s**	**0.1 mm/s**	**0.2 mm/s**	**0.3 mm/s**	**0.4 mm/s**	**0.5 mm/s**	**1 mm/s**
**RMSE**	0.0633	0.0766	0.0797	0.0820	0.0882	0.0883	0.0887	0.0896
**R^2^**	0.9968	0.9957	0.9957	0.9952	0.9957	0.9954	0.9959	0.9963
**Hysteresis**	5.75%	6.31%	6.36%	6.30%	7.56%	8.41%	8.87%	11.43%

**Table 3.** Mean and standard deviation of maximum and average force recorded by right-leg thigh cuff front- and back-side PSPs 2.1 during a gait cycle. Data are reported for the three different gait conditions (*i.e.*, gait velocity equal to 4 km/h, with and without assistive torque, and gait velocity equal to 2.5 km/h with assistive torque) and the three subjects. Maximum and average force values are averaged over 20 gait cycles.

	**Maximum force [N]**	**Average force [N]**	**Maximum force [N]**	**Average force [N]**
	***Front-side***		***Back-side***	
**Gait velocity: 4 km/h, assistance: NO**				
**Subject #1**	27.40 ± 1.62	12.79 ± 0.49	49.24 ± 5.07	28.62 ± 2.20
**Subject #2**	21.22 ± 1.58	11.38 ± 0.40	22.54 ± 3.96	10.06 ± 0.70
**Subject #3**	20.27 ± 1.44	10.79 ± 0.27	19.82 ± 2.53	9.92 ± 0.56
**Gait velocity: 4 km/h, assistance: YES**				
**Subject #1**	25.72 ± 0.74	14.61 ± 0.33	49.25 ± 6.18	23.80 ± 1.04
**Subject #2**	22.18 ± 0.67	12.13 ± 0.22	17.83 ± 1.97	9.34 ± 0.39
**Subject #3**	20.51 ± 0.85	11.59 ± 0.30	17.01 ± 1.81	8.82 ± 0.28
**Gait velocity: 2.5 km/h, assistance: YES**				
**Subject #1**	19.64 ± 1.08	13.42 ± 0.62	29.23 ± 4.00	21.46 ± 1.13
**Subject #2**	14.62 ± 0.84	9.95 ± 0.23	13.94 ± 1.60	8.35 ± 0.48
**Subject #3**	14.27 ± 0.46	10.27 ± 0.56	12.40 ± 2.54	7.70 ± 0.35

## References

[1] Turchetti B.G., Micera S., Cavallo F., Odetti L., Dario P. (2011). Technology and innovative services. IEEE Pulse.

[2] Leven J., Burschka D., Kumar R., Zhang G., Blumenkranz S., Dai X., Award M., Hager G.D., Marohn M., Choti M. (2005). DaVinci Canvas: A telerobotic surgical system with integrated, robot-assisted, laparoscopic ultrasound capability. Med. Image Comput. Comput. Assist. Interv..

[3] Masia L., Krebs H.I., Cappa P., Hogan N. (2007). Design and characterization of hand module for whole-arm rehabilitation following stroke. IEEE/ASME Trans. Mechatr..

[4] Jezernik S., Colombo G., Keller T., Frueh H., Morari M. (2003). Robotic orthosis lokomat: A rehabilitation and research tool. Neuromodulation.

[5] Volpe B.T., Huerta P.T., Zipse J.L., Rykman A., Edwards D., Dipietro L., Hogan N., Krebs H.I. (2009). Robotic devices as therapeutic and diagnostic tools for stroke recovery. Arch. Neurol..

[6] Guizzo E., Goldstein H. (2005). The rise of the body bots. IEEE Spectr..

[7] Banala S., Agrawal S., Scholz J. (2009). Robot assisted gait training with active leg exoskeleton (ALEX). IEEE Trans. Neur. Syst. Rehab. Eng..

[8] Schiele A., Visentin G. The ESA Human Arm Exoskeleton for Space Robotics Telepresence.

[9] Walsh C., Pasch K., Herr H. An Autonomous, Underactuated Exoskeleton for Load-Carrying Augmentation.

[10] Pylatiuk C., Kargov A., Gaiser I., Werner T., Schulz S., Bretthauer G. Design of a Flexible Fluidic Actuation System for a Hybrid Elbow Orthosis.

[11] Kong K., Jeon D. (2006). Design and control of an exoskeleton for the elderly and patients. IEEE/ASME Trans. Mechatr..

[12] Suzuki K., Mito G., Kawamoto H, Hasegawa Y., Sankai Y. (2007). Intention-based walking support for paraplegia patients with robot suit HAL. Adv. Robot..

[13] Zoss A.B., Kazerooni H., Chu A. (2006). Biomechanical design of the Berkeley lower extremity exoskeleton (BLEEX). IEEE ASME Trans. Mechatr..

[14] Lünenburger L., Colombo G., Riener R., Dietz V. Clinical Assessments Performed During Robotic Rehabilitation by the Gait Training Robot Lokomat.

[15] Dollar A.M., Herr H. (2008). Lower Extremity Exoskeletons and Active Orthoses: Challenges and State-of the-Art. IEEE Trans. Robot..

[16] Pons J.L. (2008). Wearable Robots: Biomechatronic Exoskeletons.

[17] Pons J.L. (2010). Rehabilitation exoskeletal robotics. IEEE Eng. Med. Biol. Mag..

[18] De Santis A., Siciliano B., De Luca A., Bicchi A. (2008). An atlas of physical human-robot interaction. Mech. Mach. Theory.

[19] Veneman J.F., Kruidhof R., Hekman E.E., Ekkelenkamp R., Van Asseldonk E.H., van der Kooij H. (2007). Design and evaluation of the LOPES exoskeleton robot for interactive gait rehabilitation. IEEE Trans. Neur. Syst. Rehab. Eng..

[20] Stienen A., Hekman E., van der Helm F., Prange G., Jannink M., Aalsma A., van der Kooij H. Dampace: Dynamic Force-Coordination Trainer for the Upper Extremities.

[21] Mihelj M., Nef T., Riener R. ARMin II—7 DoF Rehabilitation Robot: Mechanics and Kinematics.

[22] Dollar A.M., Herr H. Design of a Quasi-Passive Knee Exoskeleton to Assist Running.

[23] Allemand Y., Stauffer Y., Clavel R., Brodard R. Design of a New Lower Extremity Orthosis for Overground Gait Training with the WalkTrainer.

[24] Vitiello N., Lenzi T., Roccella S., De Rossi S.M.M., Cattin E., Giovacchini F., Vecchi F., Carrozza M.C. (2012). NEUROExos: A powered elbow exoskeleton for physical rehabilitation. IEEE Trans. Robot..

[25] Chiri A., Vitiello N., Giovacchini F., Roccella S., Vecchi F., Carrozza M.C. (2012). Mechatronic design and characterization of the index finger module of a hand exoskeleton for post-stroke rehabilitation. IEEE Trans. Mechatr..

[26] Kao P.-C., Ferris D.P. (2009). Motor adaptation during dorsiflexion-assisted walking with a powered orthosis. Gait Posture.

[27] Ronsse R., Vitiello N., Lenzi T., van den Kieboom J., Carrozza M.C., Ijspeert A.J. (2011). Human-robot synchrony: Flexible assistance using adaptive oscillators. IEEE Trans. Biomed. Eng..

[28] Rocon E., Ruiz A.F., Manto M., Moreno J.C., Pons J.L. (2007). Design and validation of a rehabilitation robotic exoskeleton for tremor assessment and suppression. Trans. Neur. Syst. Rehab. Eng..

[29] Beyl P., Van Damme M., Van Ham R., Versluys R., Vanderborght B., Lefeber D. An Exoskeleton for Gait Rehabilitation: Prototype Design and Control Principle.

[30] Kim K., Hong K.-J., Kim N.-G., Kwon T.-K. (2011). Assistance of the elbow flexion motion on the active elbow orthosis using muscular stiffness force feedback. J. Mech. Sci. Technol..

[31] Tanada T., Hori S., Yamaguchi R., Feng M.Q. Ultrasonic Sensor Disk for Detecting Muscular Force.

[32] Lee H., Yu S., Lee S., Han J., Han C. Development of Human-Robot Interfacing Method for Assistive Wearable Robot of the Human Upper Extremities.

[33] Lenzi T., Vitiello N., De Rossi S.M.M., Persichetti A., Giovacchini F., Roccella S., Vecchi F., Carrozza M.C. (2011). Measuring human-robot interaction on wearable robot: A distributed approach. Mechatronics.

[34] De Rossi S.M.M., Vitiello N., Lenzi T., Ronsse R., Koopman B., Persichetti A., Giovacchini F., Vecchi F., Ijspeert A.J., van der Kooij H., Carrozza M.C. Soft Artificial Tactile Sensors for the Measurement of Human-Robot Interaction in the Rehabilitation of the Lower Limb.

[35] Gonzalez J., Garcýa A., Vivas M., Ferrus E., Alcantara E., Forner A. A New Portable Method for the Measurement of Pressure Discomfort Threshold (ptd) on the Foot Plant.

[36] Krouskop T.A., Williams R., Krebs M., Herszkowicz M.S., Garber S. (1985). Effectiveness of mattress overlays in reducing interface pressures during recumbency. J. Rehab. Res. Dev..

[37] De Rossi S.M.M., Vitiello N., Lenzi T., Ronsse R., Koopman B., Persichetti A., Vecchi F., Ijspeert A.J., van der Kooij H., Carrozza M.C. (2011). Sensing pressure distribution on a lower-limb exoskeleton physical human-machine interface. Sensors.

[38] Lenzi T., Vitiello N., De Rossi S.M. M, Roccella S., Vecchi F., Carrozza M.C. NEUROExos: A Variable Impedance Powered Elbow Exoskeleton.

[39] De Rossi S.M.M., Lenzi T., Vitiello N., Donati M., Persichetti A., Giovacchini F., Vecchi F., Carrozza M.C. Development of an In-Shoe Pressure Sensitive Device for Gait Analysis.

[40] Persichetti A., Vecchi F., Carrozza M.C. (2009). Conformant and flexible tactile sensor and method therefore.

[41] Persichetti A., Vecchi F., Vitiello N., Lenzi T., Carrozza M.C. Skilsens: Conformant and Robust Sensing Skin.

[42] Miller K. (2000). Testing Elastomers for Hyperelastic Material Models in Finite Elements Analysis.

[43] Pearson I., Pickering M. (2001). The determination of a highly elastic adhesive's material properties and their representation in finite element analysis. Finite Elem. Anal. Design.

[44] Miller K. (2006). Measuring Rubber and Plastic Friction for Analysis.

[45] De Rossi S.M.M., Lenzi T., Vitiello N., Persichetti A., Giovacchini F., Carrozza M.C. Struttura di tappeto sensorizzato (Sensorized mat structure).

[46] De Rossi S.M.M., Lenzi T., Vitiello N., Persichetti A., Giovacchini F., Carrozza M.C. Structure of Sensorized mat.

[47] Perry J. (1992). Gait Analysis: Normal and Pathological Function.

[48] Veneman J.F., Ekkelenkamp R., Kruidhof R., van der Helm F.C.T., van der Kooij H. (2006). A series elastic- and Bowden-cable-based actuation system for use as torque actuator in exoskeleton-type robots. Int. J. Robot. Res..

[49] Ekkelenkamp R., Veltink P., Stramigioli S., van der Kooij H. Evaluation of a Virtual Model Control for Selective Support of Gait Functions Using an Exoskeleton.

[50] De Rossi S.M.M., Crea S., Donati M., Reberšek P., Novak D., Vitiello N., Lenzi T., Podobnik J., Munih M., Carrozza M.C. Gait Segmentation Using Bipedal Foot Pressure Patterns.

[51] Crea S., De Rossi S.M.M., Donati M., Reberšek P., Novak D., Vitiello N., Lenzi T., Podobnik J., Munih M., Carrozza M.C. Development of Gait Segmentation Methods for Wearable Foot Pressure Sensors.

[52] Ronsse R., Lenzi T., Vitiello N., Koopman B., van Asseldonk E., De Rossi S.M.M., van den Kieboom J., van der Kooij H., Carrozza M.C., Ijspeert A.J. (2011). Oscillator-based assistance of cyclical movements: Model-based and model-free approaches. Med. Biol. Eng. Comput..

[53] Crea S., Vitiello N., De Rossi S.M. M., Lenzi T., Donati M., Cipriani C., Carrozza M.C. Development of an Experimental Set-Up for Providing Lower-Limb Amputees with an Augmenting Feedback.

[54] Harada T., Sato T., Mori T. Estimation of Bed-Ridden Human's Gross and Slight Movement Based on Pressure Sensors Distribution Bed.

[55] Fallang B., Saugstad O.D., Hadders-Algra M. (2003). Postural adjustments in preterm infants at 4 and 6 months post-term during voluntary reaching in supine position. Pediatr. Res..

